# Small Glutamine-Rich Tetratricopeptide Repeat-Containing Protein Alpha (SGTA) Ablation Limits Offspring Viability and Growth in Mice

**DOI:** 10.1038/srep28950

**Published:** 2016-06-30

**Authors:** Lisa K. Philp, Tanya K. Day, Miriam S. Butler, Geraldine Laven-Law, Shalini Jindal, Theresa E. Hickey, Howard I. Scher, Lisa M. Butler, Wayne D. Tilley

**Affiliations:** 1 Adelaide Prostate Cancer Research Centre and Dame Roma Mitchell Cancer Research Laboratories, Faculty of Health Sciences, University of Adelaide, Adelaide, Australia; 2 Memorial Sloan Kettering Cancer Center, New York, NY, USA; 3 Freemason’s Foundation Centre for Men’s Health, School of Medicine, Faculty of Health Sciences, University of Adelaide, Adelaide, Australia

## Abstract

Small glutamine-rich tetratricopeptide repeat-containing protein α (SGTA) has been implicated as a co-chaperone and regulator of androgen and growth hormone receptor (AR, GHR) signalling. We investigated the functional consequences of partial and full *Sgta* ablation *in vivo* using Cre-lox *Sgta*-null mice. *Sgta*^+/−^ breeders generated viable *Sgta*^−/−^ offspring, but at less than Mendelian expectancy. S*gta*^−/−^ breeders were subfertile with small litters and higher neonatal death (*P* < 0.02). Body size was significantly and proportionately smaller in male and female *Sgta*^−/−^ (vs WT, *Sgta*^+/−^ *P* < 0.001) from d19. Serum IGF-1 levels were genotype- and sex-dependent. Food intake, muscle and bone mass and adiposity were unchanged in *Sgta*^−/−^. Vital and sex organs had normal relative weight, morphology and histology, although certain androgen-sensitive measures such as penis and preputial size, and testis descent, were greater in *Sgta*^−/−^. Expression of *AR* and its targets remained largely unchanged, although AR localisation was genotype- and tissue-dependent. Generally expression of other TPR-containing proteins was unchanged. In conclusion, this thorough investigation of SGTA-null mutation reports a mild phenotype of reduced body size. The model’s full potential likely will be realised by genetic crosses with other models to interrogate the role of SGTA in the many diseases in which it has been implicated.

Small glutamine-rich tetratricopeptide repeat (TPR)-containing protein α (SGTA) belongs to a family of molecular co-chaperone proteins that possess a TPR motif. This motif, when arrayed in tandem, forms a protein-protein interaction module capable of facilitating interactions with a diverse range of client proteins (as reviewed in refs 1, 2, 3). Since its discovery 15 years ago^4^, SGTA has been implicated in a myriad of biological processes^1^ including cell cycle and apoptosis^5^, viral assembly and release^4^,^6^,^7^,^8^,^9^, hormone signalling^5^,^10^,^11^,^12^,^13^,^14^,^15^,^16^,^17^, intracellular compartmentalization^10^,^11^, neuronal synaptic transmission^18^ and the post-translational transport^19^,^20^,^21^,^22^ and modification^17^,^23^ of proteins. Due to its broad pattern of tissue expression^1^,^24^,^25^ (Suppl Fig. 1A,B) and its engagement in many biological processes, SGTA has been implicated in many diseases including cancer of the prostate^10^,^26^, ovary^27^, liver^28^ and oesophagus^29^; hormone-related polycystic ovary syndrome^30^; and amyloid-related Alzheimer’s^18^ and prion^31^ diseases. Given the overarching cellular role of SGTA in co-chaperoning client proteins to ensure their correct folding, trafficking and/or appropriate subcellular movement in the aforementioned biological processes^1^, it is surprising that its essential normal biological functions remain uncharacterised.

Throughout evolution SGTA, and in particular its TPR motif, has remained conserved^4^,^6^,^10^,^11^,^14^,^32^, with orthologs to human *Sgta* detected in *Caenorhabditis elegans*^32^*, Drosophila melanogaster*^33^ and *Saccharomyces cerevisiae*^32^. SGTA is ubiquitously expressed in human and murine tissues, with *M. musculus* SGTA exhibiting 83% and 88% identity to human SGTA at the mRNA and protein levels, respectively, with similar distribution of functional domains (Suppl Fig. 1D–F). Knockdown of *SGTA in vitro* can impair cellular proliferation (NBE, HeLa, C4-2B cells), reduce cell viability (C4-2B cells)^26^ and promote cell death (NBE, HeLa cells)^5^,^34^. No study to date has reported the effect of SGTA ablation *in vivo*. Given that SGTA is expressed in early development^35^ and has a ubiquitous co-chaperoning role, we aimed to determine the physiological importance of SGTA *in vivo* by assessing the effect of SGTA ablation on normal development and fecundity in mice.

## Results

### *Sgta* ^−/−^ Mice are Viable but Tended to Exhibit Reduced Perinatal Viability

*Sgta*^+/−^ breeders were overtly normal in appearance and were fertile, with time to litter comparable to *Sgta-*intact animals, and viable offspring representing all possible genotypes (*Sgta*^−/−^, *Sgta*^+/−^ and WT) were produced (Table 1). Gross phenotypic inspection of offspring indicated that all were visibly normal. *Sgta*^+/−^ breeders yielded litters of normal size, with unchanged neonatal mortality rate, as compared to *Sgta*-intact litters (Table 1). However, the Mendelian ratio of inheritance was altered in progeny from *Sgta*^+/−^ matings; irrespective of gender, *Sgta*^−/−^ pups tended to be less frequently observed than the expected ratio (22.49% vs expected 25%, *P* = 0.095). However, when separated by sex this was most pronounced for female *Sgta*^−/−^ pups (18.80% vs 25.76% for males, compared to expected 25%), suggesting that female *Sgta*^−/−^ mice were less viable during the perinatal period. Sex ratios did not deviate from normal (1.06M: 0.94F vs expected 1M: 1F, *P* = 0.38).

### *Sgta* Knockout was Complete and Global

**Figure 1:**
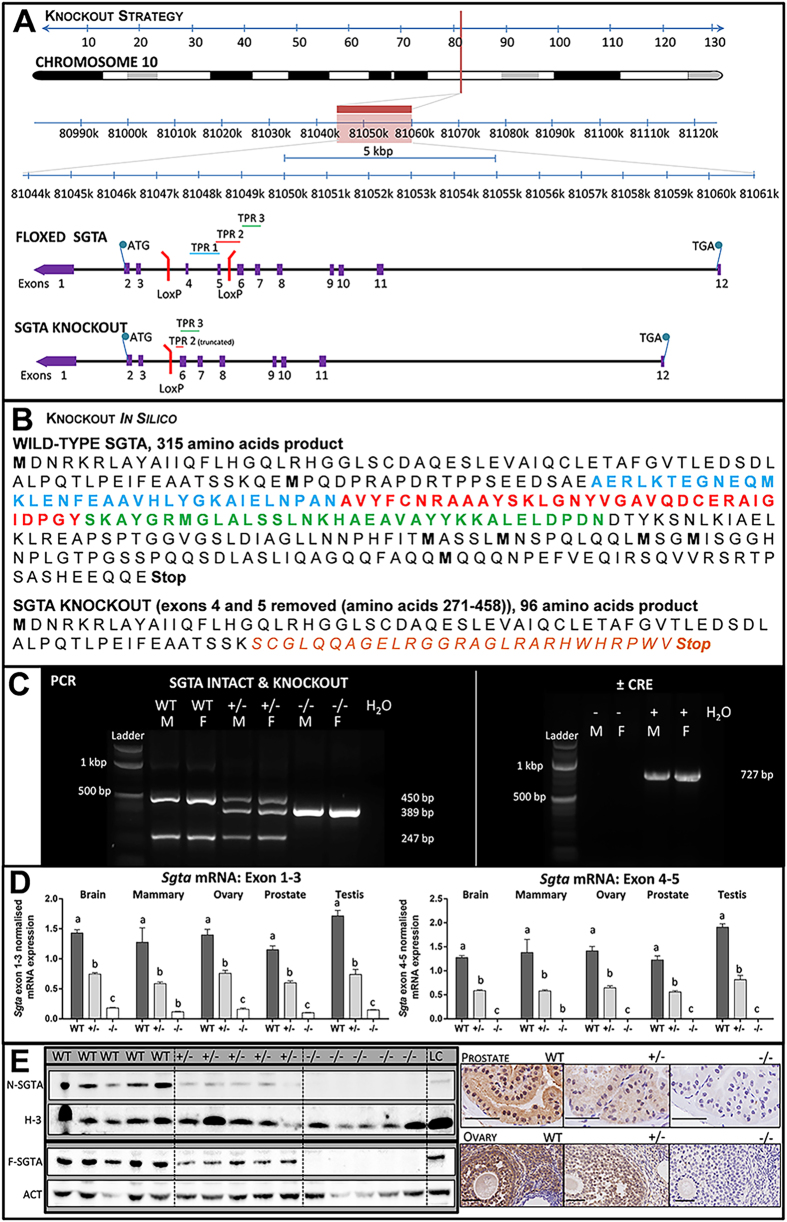
Generation of Mice Null for Small Glutamine-rich Tetratricopeptide Repeat-containing Protein Alpha (SGTA). (**A**) Genomic structure of the mouse *Sgta* gene floxed at exons 4 and 5, and schematic of the resultant knockout product. (**B**) Protein sequence of wild-type SGTA, with TPR1, 2 and 3 highlighted in blue, red and green, respectively, and effect of exons 4–5 (amino acids 271–458) deletion leading to a 96 amino acid product. (**C**) PCR analyses confirming *Sgta* deletion in heterozygous (+/−) and homozygous (−/−) *Sgta*-null mice using ‘SGTA knockout’ primers, and the presence of *Sgta* in wild-type (WT) and *Sgta* heterozygous (+/−) mice, using ‘SGTA intact’ primers and finally the removal of Cre Recombinase (*Cre*) in a subset of *Sgta*^+/−^ mice (L, ladder; bp, base pairs; H_2_O, water control; M, male; F, female; N.B. ladder’s two brightest bands at 1000 (upper) and 500 (lower) bp). (**D**) Quantitative Real Time PCR confirming homozygous *Sgta* mutation ablated *Sgta* mRNA compared to *Sgta*^+/−^ and WT, while heterozygous *Sgta* mutation significantly diminished *Sgta* mRNA compared to WT. Analyses were conducted in brain, mammary, ovary, prostate, and testis tissues with primers spanning in either *Sgta* exon 1–3 and *Sgta* exon 4–5. Means (n = 8/group) with different letters are significantly different, *P* < 0.001. (**E**) Western blots (brain) and immunohistochemistry (prostate, ovary) confirming loss of SGTA protein in homozygous *Sgta*-null (−/−) tissue and lowered SGTA protein expression in heterozygous *Sgta*-null (+/−) tissue (LC, loading control; N-SGTA, probed with ‘N-terminal’ SGTA antibody; F-SGTA, probed with ‘Full length’ SGTA antibody; H-3, Histone H-3; ACT, Actin). For Western blots, representative cropped blots are depicted, with all blots run under the same experimental conditions. Scale bars represent 50μm.

Having determined that *Sgta*^−/−^ mice are viable, we next characterised the functional consequence of the *Sgta* disruption. The Cre/Lox knockout strategy used in this study (Fig. 1A) involved floxing exons 4 and 5 of *M. musculus Sgta* for disruption by *Cre*. *In silico* analysis of the knockout strategy demonstrated that in the case that a stable mRNA species was transcribed, SGTA would be truncated at the TPR domain, generating a novel 27 amino acid sequence, followed by a stop codon, theoretically producing a 96 amino acid-long protein devoid of TPR-motifs (Fig. 1B). Efficiency of deletion was confirmed by PCR (Fig. 1C). At the mRNA level, *Sgta* abundance was assessed by qRT-PCR using *Sgta* exon1–3 (‘N-terminal’) and *Sgta* exon4–5 (‘Knockout’) primers in the brain, mammary, ovary, prostate and testis. ‘Knockout’ primers failed to amplify product and *Sgta* knockout mRNA levels were dose-dependent for *Sgta* WT, ^+/−^ and ^−/−^ tissues (*P* < 0.001; Fig. 1D). Homozygous *Sgta*-null mice exhibited severely blunted *Sgta* ‘N-terminal’ mRNA expression. Again, ‘N-terminal’ *Sgta* mRNA levels were dose-dependent (*P* < 0.001; Fig. 1D). These data demonstrate that this strategy resulted in mice completely lacking *Sgta* mRNA, rather than a functional knockout. Complete ablation of SGTA protein was confirmed by Western blot analyses (Fig. 1E), but was apparent in a dose-dependent manner in WT and *Sgta*^+/−^ as expected. In the event that a truncated protein was translated following deletion of *Sgta* exon4–5, its predicted weight would be ~10.6 kDa. Even when protein was subjected to gel electrophoresis using high percentage gels specially designed to enable separation of low molecular weight proteins (as low as 2kDa), no ~10kDa bands were detected (data not shown). The complete lack of SGTA protein was confirmed by immunohistochemistry in prostate and ovary (Fig. 1E) using an antibody raised against ‘N-terminal’ SGTA. We therefore conclude that ablation of SGTA protein in these mice was both global and complete.

### Full, but not Partial, *Sgta* Deficiency Elicits a Phenotype of Reduced Body Size

**Figure 2:**
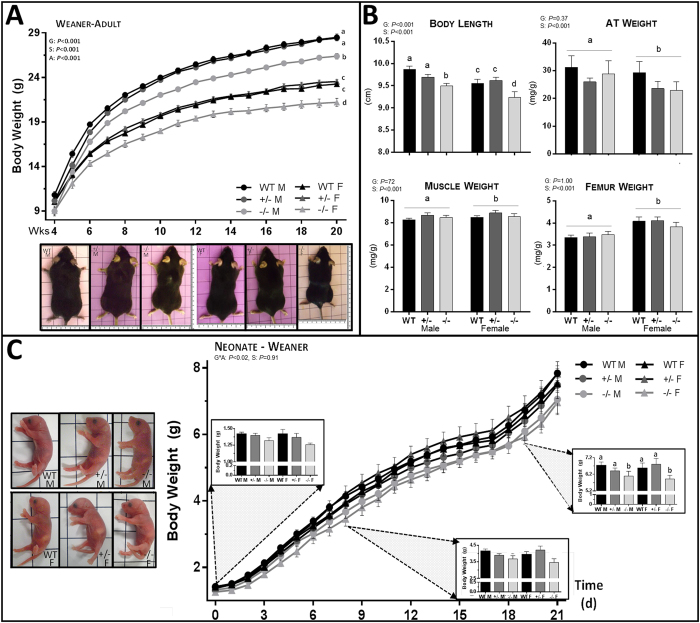
Morphological Analysis of *Sgta*-deficient Male and Female Mice at Adult and Neonatal Time Points. (**A**) Body weight growth curves from weaning to adulthood (n = males (M), WT = 23–32, +/− = 45–57, −/− = 19–24; females (F), WT = 24–34, +/− = 36–49; −/− = 10–14) and representative images of adult male and female WT, *Sgta*^+/−^ and *Sgta*^−/−^ mice. (**B**) Adult body length (n = 8–15/group) and the mass of pooled adipose tissue (AT) (n = 7–10/group), hind limb skeletal muscles (n = 7–10/group) and the femur (n = 6–9/group) relative to body weight. (**C**) Body weight growth curves from birth to weaning (n = M, F WT = 8, 6, +/− = 8, 8 −/− = 9, 5) and representative images of neonatal male and female WT, *Sgta*^+/−^ and *Sgta*^−/−^ mice. (**A**–**D**) Statistics: N.B. G, genotype; A, age, S, sex, *denotes an interaction. Means with different letters are significantly different, *P* < 0.05.

From weaning through to adulthood (20–60 wks), and irrespective of gender, *Sgta*^−/−^ mice weighed significantly less than their *Sgta*^+/−^ and WT littermates (*P* < 0.001; Fig. 2A). Body length was reduced in *Sgta*^−/−^ adult males and females as compared to *Sgta*^+/−^ and WT littermates (*P* < 0.001), confirming that *Sgta*^−/−^ mice were proportionately smaller (Fig. 2B). Reduced adiposity did not contribute to lower body mass in *Sgta*^−/−^ adults, as neither the absolute nor relative mass of 4 pooled adipose tissue depots were affected by genotype (Fig. 2B). Adipose tissue cellularity was also unaffected at the microscopic level, as assessed by a pathologist (Suppl Fig. 2). Changes in lean and skeletal mass are logical contributors for this size difference. However, pooled absolute hind limb muscle mass, whilst significantly reduced in *Sgta*^−/−^ adult mice vs *Sgta*^+/−^ and WT (*P* < 0.01), was unchanged when expressed relative to body weight (Fig. 2B). Skeletal muscle was also histologically normal across genotypes (Suppl Fig. 2). Likewise, absolute femur weight, whilst significantly reduced in *Sgta*^−/−^ vs WT adults (*P* < 0.03), when corrected for body weight, was unchanged (Fig. 2B). Therefore changes in lean and skeletal mass are unlikely to account for smaller body mass.

**Figure 3:**
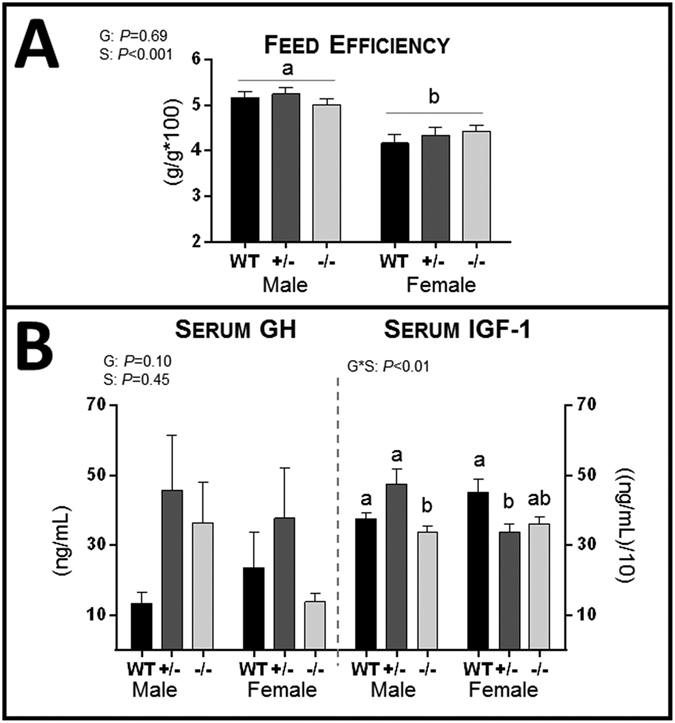
Analysis of Growth Mediators in Adult *Sgta*-deficient Male and Female Mice. (**A**) Feed efficiency (n = M, F WT = 8, 6 +/− = 8, 8 −/− = 9, 5). (**B**) Serum concentrations (n = 6/group) of growth hormone (GH) and insulin-like growth factor 1 (IGF-1). (**A**,**B**) Statistics: N.B. G, genotype; A, age, S, sex, *denotes an interaction. Means with different letters are significantly different, *P* < 0.05.

We next aimed to determine the timing and cause of body weight divergence as a result of *Sgta* ablation. *Sgta*-null neonates appeared macroscopically indistinguishable from WT, displaying no signs of disadvantage (Fig. 2C). Measuring pup weight from birth to weaning (Fig. 2C) revealed a genotype*age interaction (*P* < 0.02). From postnatal d0-7, *Sgta*^−/−^ pups exhibited similar body weight to *Sgta*^+/−^ and WT littermates. While body weights were monitored from birth, it was not until day 19 onwards that *Sgta*^−/−^ pup weight diverged significantly from *Sgta*^+/−^ and WT animals (*P* < 0.02; Fig. 2C **inset**). Feed intake did not account for smaller body mass as feed efficiency, measured from postnatal d21-105 was unchanged by genotype (Fig. 3A) and during early neonatal life, milk bands were clearly visible (Fig. 2C **inset**).

### Effects of *Sgta* Ablation on Endocrine Growth Mediators

Serum growth hormone (GH) levels were highly variable and did not significantly differ by genotype (Fig. 3B), but serum levels of another growth mediator, IGF-1^36^, were genotype- and sex-dependent (Genotype*Sex: *P* < 0.01). In males, serum IGF-1 levels were lower in *Sgta*^−/−^ than *Sgta*^+/−^ and WT (*P* < 0.01; Fig. 3B). In females, serum IGF-1 was lower in *Sgta*^+/−^ (*P* < 0.02), but not *Sgta*^−/−^ mice, compared to WT (Fig. 3B). mRNA expression of the receptors responsible for GH and IGF-1 action, growth hormone receptor (*Ghr*) and *Igf-1* receptor (*Igf-1r*), and for *Igf-1* transcript were assessed by qRT-PCR in tissues. In the ovary, *Ghr* mRNA was higher in *Sgta*^−/−^ than WT (*P* < 0.03), but levels were unchanged by genotype in the mammary, prostate or testis (Table 2). *Igf-1* mRNA levels were significantly lower in the mammary gland of *Sgta*^−/−^ females than *Sgta*^+/−^ (*P* < 0.05; Table 2). In testis, *Igf-1* mRNA was unchanged with genotype, as were *Igf-1r* mRNA levels in the ovary, mammary, prostate and testis (Table 2).

### *Sgta* ^−/−^ Breeders Bear Offspring with Reduced Perinatal Viability

As *Sgta*-null mice were viable, male and female *Sgta*^−/−^ mice were paired to assess fecundity. Subfertility and reduced pup viability were observed in this cohort (Table 1). Litter size trended smaller when from *Sgta*^−/−^ breeding pairs, vs litters from *Sgta*^+/−^ (*P* < 0.06) breeders. Delayed time to litter with decreasing *Sgta* ‘dose’ (*Sgta*-WT > *Sgta*^+/−^ > *Sgta*^−/−^) failed to reach significance (*P* = 0.17). Offspring were no more susceptible to stillbirth, but were more prone to neonatal death between postnatal d2-21 and markedly less mice survived to weaning in *Sgta*^−/−^ vs *Sgta*-intact (*P* < 0.02) and *Sgta*^+/−^ (*P* < 0.01). Sex ratios in *Sgta*^−/−^ litters did not deviate from normal (0.94M:1.06F vs expected 1:1, *P* = 0.72).

### Effect of *Sgta* Ablation on Reproductive Organ Development and AR Signalling

**Figure 4:**
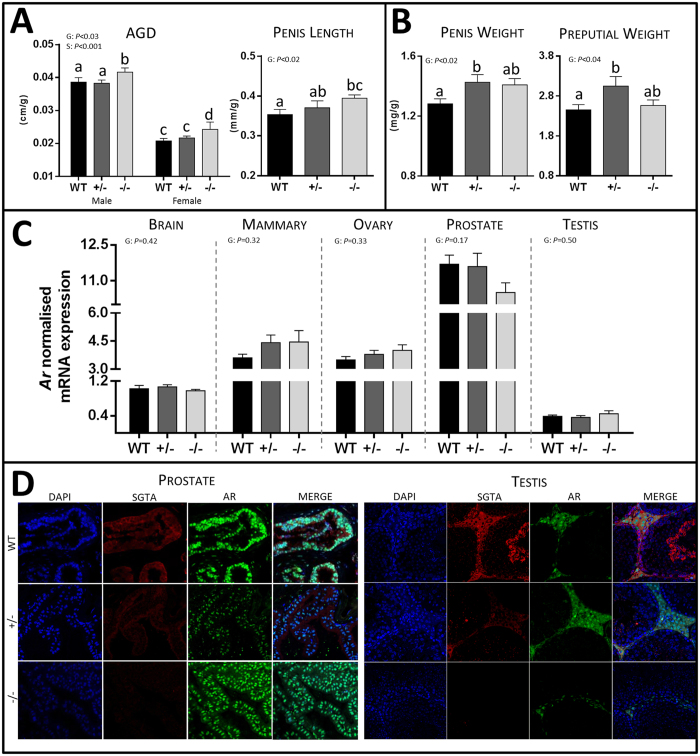
The Effect of Full and Partial SGTA Deficiency on Androgen-Regulated Measures and AR Signalling. (**A**) Androgen-regulated developmental measures including anogenital distance (AGD; n = 12–15/group, bar *Sgta*^−/−^ F, n = 7) and penis length in WT, *Sgta*^+/−^ and *Sgta*^−/−^ adult mice. (**B**) Androgen-regulated developmental measures including penis and preputial gland weight (n = 7–9/group) in WT, *Sgta*^+/−^ and *Sgta*^−/−^ adult mice. (**C**) *Ar* mRNA expression in the brain, mammary, ovary, prostate and testis of WT, *Sgta*^+/−^ and *Sgta*^−/−^ adult mice (n = 7–8/group). (**D**) Representative images of AR (green) and SGTA (red) protein co-localisation in the prostate and testis of WT, *Sgta*^+/−^ and *Sgta*^−/−^ adult mice. Statistics: N.B. G, genotype; S, sex. Means with different letters are significantly different, *P* < 0.05.

SGTA has been implicated as a putative co-chaperone for the AR and is believed to limit translocation of AR to the nucleus until the AR complex is ligand-bound^10^. Androgen and AR signalling pathways are involved in the development of the male-specific phenotype during early development and in spermatogenesis, sexual behaviour and fertility in male adult life, but are also important in female sex organ development, including uterus and breast, and normal reproductive physiology, such as ovarian folliculogenesis and embryonic implantation, in females. Chemical or genetic disruption of androgen/AR signalling perturbs normal male and female reproductive development or function^37^,^38^. We therefore assessed AGD (testis descent) and penis and preputial size as surrogates for androgen/AR signalling during early prenatal, prepubertal^37^,^38^ and pubertal^39^,^40^ development, respectively (Fig. 4A,B).

In general, the phenotypic effects of SGTA deficiency on the male reproductive system were subtle. The most significant effect was on testis descent, as AGD was significantly increased in *Sgta*^−/−^ males compared to other males (WT and *Sgta*^+/−^; *P* < 0.001; Fig. 4A). Penis length was increased in *Sgta*^−/−^ vs WT males (*P* < 0.02; Fig. 4A), and penis weight was greater in *Sgta*^+/−^ (*P* < 0.05), and trended higher in *Sgta*^−/−^ (*P* = 0.062), vs WT (Fig. 4B). Similarly, preputial gland weight was greater in *Sgta*^+/−^ vs WT males (*P* < 0.05; Fig. 4B). However, other male reproductive organs were unchanged as a result of homozygous *Sgta* ablation in their mass (Suppl Fig. 3A), macroscopic appearance (Suppl Fig. 3B) and pathology (Suppl Fig. 2) when compared to WT. *Sgta*^−/−^ males also exhibited sperm in both the testes, the site of spermatogenesis^41^, and epididymis, the site of sperm maturation and storage^42^. Similarly, female reproductive organs were unchanged as a result of homozygous *Sgta* ablation in their mass (Suppl Fig. 3C), morphology (Suppl Fig. 3D) or histology (Suppl Fig. 2) when compared to WT. The ovaries, which depend on androgens for normal function and early follicular development^38^, were phenotypically normal at the macro- (Suppl Fig. 3C), and microscopic levels in *Sgta*^−/−^ females as assessed by a pathologist (Suppl Fig. 2). Ovaries also exhibited follicles at all stages of follicular development^43^, including primordial, primary, secondary and antral follicles, and the corpus luteum.

As some, but not all, androgen-regulated organs displayed potential signs of hyperandrogenisation during development in *Sgta*^+/−^ and *Sgta*^−/−^ mutants, we measured *Ar* mRNA expression in androgen-regulated tissues, the brain, and the prostate and testis in males, and the mammary and ovary in females (Fig. 4C). *Ar* mRNA was unchanged by genotype in the prostate, testis, ovary, mammary and brain. To determine if *Sgta* ablation altered the localisation and distribution of AR protein, we undertook dual-labelled immunofluorescence staining of prostate and testis sections (Fig. 4D). In the prostate, SGTA protein resided chiefly in the cytoplasm of WT and *Sgta*^+/−^ epithelial cells, with weaker intensity staining in *Sgta*^+/−^ than WT, and no staining in *Sgta*^−/−^ animals, as expected (Fig. 4D). Conversely, AR protein was principally nuclear in WT and *Sgta*^+/−^ prostate epithelial cells, with weaker intensity staining in *Sgta*^+/−^ vs WT. However, in *Sgta*^−/−^ prostates, AR immunostaining was equally as intense as in the nuclei of WT prostate, but exhibited greater immunoreactivity in the cytoplasm than WT and *Sgta*^+/−^. In the testis, SGTA was also localised predominantly in the cytoplasm of cells and was detected in the Leydig, Sertoli, spermatid and spermatocyte cells of WT testis (Fig. 4D). AR immunostaining was detected predominantly within the Leydig cells, with intense nuclear and distinct cytoplasmic immunoreactivity in WT testis. Partial *Sgta* ablation increased AR immunoreactivity, especially in the cytoplasm of Leydig cells, although distinct nuclear immunostaining was also detected. And finally, full *Sgta* ablation was associated with a visual reduction of AR immunoreactivity, especially in the cytoplasm, although some nuclear and cytoplasmic staining was still observed (Fig. 4D). Hence, some tissue-specific effects of SGTA dosage were evident. In order to determine if these changes were associated with changes in AR signalling, the mRNA content of known androgen-regulated target genes was assessed in the testis, prostate, ovary and mammary (Table 2). None of the AR-dependent genes measured were altered as a result of full or partial *Sgta* ablation.

### Relative to Body Weight, Organs Weights were Mostly Unchanged, Pathology was Normal and Aging was Associated with no Genotype-specific Abnormalities

As the majority of organs tended to exhibit lighter absolute weight in *Sgta*^−/−^, presumably due to their smaller body size, we assessed the effect of genotype on organ weights relative to body weight. This allowed direct comparison between genotype and gender. Other than an increased heart weight in *Sgta*^+/−^ vs WT (*P* < 0.05), brain weight in *Sgta*^−/−^ vs WT (*P* < 0.02), and intestine weight in *Sgta*^−/−^ than WT (*P* < 0.11), and a reduced relative stomach weight in *Sgta*^+/−^ vs *Sgta*^−/−^ (*P* < 0.02) and WT (*P* < 0.06), the mass of most vital organs relative to body weight was unchanged by genotype (Suppl Fig. 3E,F). All organs were also phenotypically normal in terms of morphology and pathology (Suppl Fig. 2).

Aging is considered to be a stressor and is associated with the accumulation of misfolded proteins^44^. Given the key role of SGTA in protein processing and folding^1^, we were interested to determine if aging resulted in a more pronounced phenotype in *Sgta*-null animals. However, the relative weights of vital and sex organs, adipose tissue, muscle and bone were not affected by a genotype*age interaction, suggesting that *Sgta*-null mice are not any more vulnerable to age-related stress than WT. SGTA has also been implicated in neuro-synaptic transmission^13^ and in responses to amyloid-associated toxicity^18^, however in this study *Sgta*-null animals showed no overt symptoms of neurodegeneration in either adulthood or aging.

### Other TPR-Containing Proteins are Largely Unchanged in *Sgta* ^−/−^ Mice

**Figure 5:**
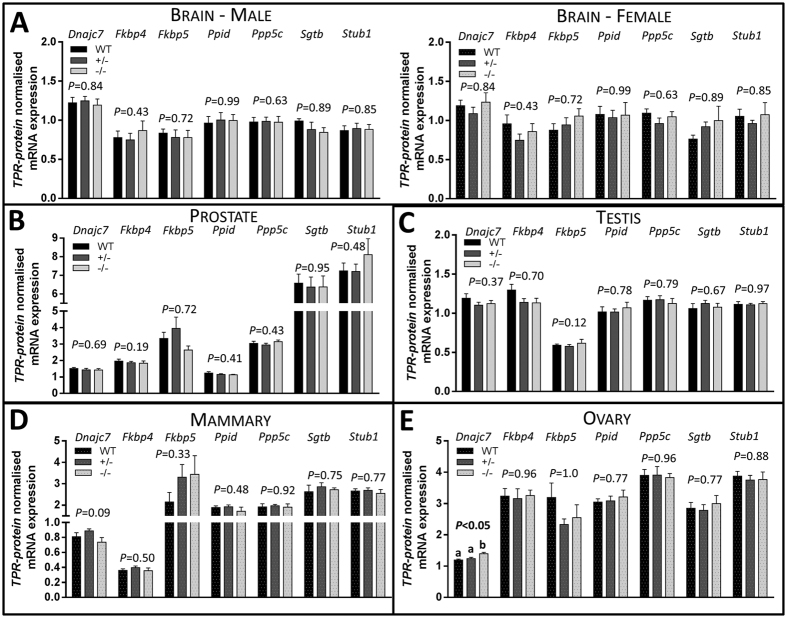
TPR-containing Protein mRNA Expression Levels in the Brain, Prostate, Testis, Mammary and Ovary of WT, *Sgta*^+/−^ and *Sgta*^−/−^ Adult Mice. TPR-containing proteins in the (**A**) brain, (**B**) prostate, (**C**) testis, (**D**) mammary and (**E**) ovary. TPR-containing proteins measured were tetratricopeptide repeat protein 2 (TPR2/*Dnajc7*), peptidyl-prolyl cis-trans isomerase FKBP4 (FKBP52/*Fkbp4*), peptidyl-prolyl cis-trans isomerase FKBP5 (FKBP51/*Fkbp5*), cyclophilin 40 (CYP40/*Ppid*), protein phosphatase 5 (PP5/*Ppp5c*), small glutamine-rich tetratricopeptide repeat-containing protein beta (SGTB/*Sgtb*) and carboxy terminus of Hsp70-interacting protein (CHIP/*Stub1*) (n = 7–9/group). Statistics: Means with different letters are significantly different, *P* < 0.05.

Given the mild phenotype observed in *Sgta*^−/−^ animals, we hypothesised that one or multiple TPR-containing proteins^1^ could possibly fulfil the cellular role of SGTA and prevent catastrophic dysfunction in co-chaperone assisted pathways (Fig. 5). However, in the brain (Fig. 5A), prostate (Fig. 5B) and testis (Fig. 5C), ovary and mammary, expression of *Stub1*, *Sgtb*, *Ppp5c*, *Ppid*, *Fkbp4*, *Fkbp5* mRNAs were not affected by genotype. However, *Dnajc7* was increased in *Sgta*^−/−^ ovary compared to WT and *Sgta*^+/−^ (*P* < 0.01, *P* < 0.03, respectively; Fig. 5E).

## Discussion

Recent studies from our laboratory^10^,^26^,^27^ and others^28^,^29^, have linked altered SGTA expression to tumour cell proliferation and/or cancer prognosis. This is not surprising, given that SGTA has been implicated in cell cycle and apoptosis^5^,^45^,^46^ and in the molecular co-chaperoning of numerous protein clients to ensure their correct folding, compartmentalisation and/or trafficking^1^. Each of these processes, if defective, has the potential to contribute to tumorigenesis. Aside from a role in the pathophysiology of several disease-states^10^,^18^,^26^,^27^,^28^,^29^,^30^,^31^, little is known about the normal physiological role of SGTA.

*In vitro*, *SGTA* knockdown in kidney (NBE)^34^, cervical cancer (HeLa)^5^ and prostate cancer (C4-2B)^26^ cells reduces proliferation and viability due to impaired mitosis^5^,^34^, in association with defective cytokinesis^34^ and failed completion of chromosomal alignment^5^. Because of this, and the fact that *Sgta* is ubiquitously expressed and a wide range of protein interactions with SGTA are documented^1^, we hypothesised that *Sgta* would be vital for life and that its ablation in mice would prove lethal. On the contrary, however, we demonstrated here that viable *Sgta*^−/−^ animals were produced from breeding *Sgta*-deficient mice, as is the case in the knockout/down of conserved SGTA orthologs^32^,^33^ in *C. elegans, D. melanogaster* and *S. cerevisiae*^14^,^15^,^33^. The tendency we observed for reduced numbers of *Sgta*^−/−^ offspring in comparison to expected Mendelian inheritance is consistent with that of mutants for similar TPR-containing protein, FKBP52 (*Fkbp4*)^47^,^48^. Given considerable evidence that SGTA is involved in tail-anchored (TA) protein insertion^16^,^22^,^49^, and as TA biogenesis is essential for early development and genetic ablation disrupting this process has been linked to embryonic lethality^50^,^51^, it is possible that aberrant TA protein processing may contribute to the reduced yield of mutant animals. The cause of prenatal mortality remains undetermined, however, in the case of FKBP52^−/−^, genetic background did contribute to the penetrance of FKBP52 action. That is, independent generation of FKBP52^−/−^ mice on C57BL/6J^47^ and 129SvEv^48^ backgrounds yielded lethality at 50% and 30% of embryos from heterozygous matings, respectively. Moreover, backcrossing FKBP52-129SvEv mutants to the C57BL/6 strain amplified lethality to almost 100%^52^; highlighting the caveat that the underlying genetics of a mutant model influence the phenotype presented. Whether a more pronounced phenotype of *Sgta*^−/−^ would have been observed if it been characterised on a different background strain of mice, remains to be determined. In contrast to FKBP52^−/−^, deletion of the similar protein, FKBP51 (*Fkbp5*), had no effect on early survival and elicited no apparent altered phenotype in mice^53^, although *FKBP51* knockout (C57BL/6J background) in combination with FKPB52 deletion (129SvEv background) caused complete lethality in early embryonic life^54^. This highlights that redundancy exists in the co-chaperoning system. Consistent with this notion, FKBP52^−/−^ males developed normal testes, but displayed penile hypospadias^53^. Increased FKBP51 mRNA and protein expression in FKBP52^−/−^ testis, but not in penis, may actively prevent testis dysgenesis. In the context of homozygous *Sgta* ablation, it is possible that a structurally similar TPR-containing protein(s) intervenes, fulfilling the role of SGTA in its absence. Increased *Dnajc7* (TPR2) mRNA in the ovary may serve this purpose, however the mRNA expression of 7 TPR-containing proteins was unchanged in the prostate, testis and mammary gland. Whether changes in protein expression and/or localisation of TPR-containing protein(s) compensate for loss of SGTA in normal physiology merits further investigation and could explain why only a mild *Sgta*-null phenotype was observed in the current study. A more overt phenotype may be induced by applying a stressor to *Sgta*-null mutants, as co-chaperones are essential for optimal folding/refolding and trafficking of stress-affected client proteins^55^. In yeast null for SGTA ortholog, *Sgt-2*, two distinct cellular stressors reduced yeast viability^15^, growth and colony formation^20^ compared to wild-type yeast. This further supports that SGTA is essential under certain cellular conditions and it is likely that its loss is compensated for to prevent dysfunction when these conditions arise.

In *Sgta*-null mice, reduced body size comprising a proportionate decrease in body weight and length was the most overt phenotypic change observed and was limited to homozygous mutants. Inadequate nutrition could result in stunted *Sgta*^−/−^ growth, especially as weight differences coincided with a phase where dependence on nursing is lessened. This appears not to be the case, as feed efficiency was unchanged in *Sgta*^−/−^ mice, albeit their ability to absorb and utilise nutrients from food consumed remains unknown. Whether *Sgta* ablation prompted changes in the signalling of hormonal growth mediators warranted investigation, especially as myostatin^23^ and the GHR^12^ are SGTA client proteins. Defective development and altered mass of skeletal muscle would be predicted if *Sgta* deficiency influenced the maturation and signalling of myostatin, a negative regulator of muscle mass^56^; however neither muscle pathology nor mass were affected. GH, acting via GHR, is essential for somatic growth, cellular differentiation^57^, and paracrine production of IGF-1 36. Aberrant GHR signalling, and GH resistance, causes cessation of IGF-1 production and growth stunting. Despite both GH and GHR normally being expressed in early embryogenesis, growth changes due to altered GH signalling are only observed from postnatal d14-21 in mice^36^,^58^. Dwarfing at 3 weeks in GHR-null mice is associated with heightened GH and lowered levels of IGF-1 in the circulation^36^,^58^. Likewise, decreasing functional IGF-1R in mice (IGF-1Rneo) leads to growth stunting from 4–9 weeks of age, corresponding to a plateau in size, which is maintained^59^. Analogous to GHR/IGF-1R deficiency^58^,^59^, *Sgta*-null mutants emerged as being physically smaller than WT at ~3 weeks and plateaued at ~6 weeks, at which time females were 93%, and males 87%, of the weight of WT animals. Similar to GH/GHR, SGTA is detected prenatally in the embryo, but its functionality during early development is not known. Given its interaction with the ubiquitin-dependent endocytosis motif of precursor and mature GHR, SGTA has been speculated to prevent GHR interaction with ubiquitin machinery whilst being trafficked from the endoplasmic reticulum, where precursor GHR resides, to the cell membrane, where mature GHR functions^57^. Aberrant GHR/IGF-1R trafficking and potentially ubiquitin-mediated GHR degradation could be triggered by *Sgta* ablation. We hypothesise that this may contribute to the growth stunting observed in *Sgta*^−/−^ mice, providing the loss of SGTA is not compensated by another co-chaperone. Lowered IGF-1 serum and tissue mRNA levels in *Sgta*^−/−^ animals may reflect defective GHR signalling, whereas raised serum GH and elevated GHR in the ovary in *Sgta*^−/−^ may indicate compensation for deficient GH/IGF-1 signalling; however, the latter requires confirmation at the protein level.

Unlike homozygous FKBP52 deletion which rendered mice sterile^53^, *Sgta* deficiency elicited only mild subfertility in this study. Dysgenesis in sex organs dependent on androgen/AR-driven development were the leading cause of defective FKBP52^−/−^ male reproduction^53^, while failure of progesterone receptor-dependent uterine implantation conferred sterility in FKBP52^−/−^ females^48^. Increased surrogate measures (AGD, penis and preputial gland size) for androgen signalling *in utero*, both prepubertally and at puberty, in full or partial *Sgta* deficiency are suggestive of altered AR signalling and/or hyperandrogenisation. However, several other androgen-dependent organs were unaffected by *Sgta* ablation in males and females, as was the mRNA expression of *Ar* and AR-responsive genes. Regardless, an effect of *Sgta* ablation on sex steroid production cannot be excluded. In addition to AR regulation, SGTA also exhibits regulatory specificity for progesterone and glucocorticoid receptors^1^,^60^; thus the influence of SGTA ablation on biological processes controlled by progesterone and glucocorticoid signaling also warrant future investigation. Reduced nuclear AR immunoreactivity without a concomitant increase in the cytoplasm in the *Sgta*^+/−^ prostate, is suggestive of *Sgta*, like other TPR-containing proteins TPR2/*Dnajc7* and CHIP/*Stub1*, playing a role in AR stabilisation and/or degradation^61^. This appears to be the case for GHR^12^, where its stabilisation is not compensated for in an environment of partial *Sgta* deficiency.

In summary, this study has provided an intriguing first glimpse into the multi-faceted role of SGTA *in vivo*. Full, but not partial, ablation of SGTA conferred subfertility and limited the viability and growth of offspring. The complex interplay of molecular co-chaperones and the importance of redundancy in their physiological roles, particularly in hormone receptor maturation and signalling, is highlighted by the current findings. This *Sgta*-null model is amenable to further study of several clinical disorders, including hormone-dependent and β-amyloid diseases, where a role for SGTA has been implicated.

## Materials and Methods

### Ethics Statement

All procedures were approved by the University of Adelaide Animal Ethics Committee (M-2012-028, M-2014-050 and M-2010-016) and all studies were conducted in accordance with accepted standards of humane animal care, as outlined in the Australian Code of Practice for the Care and Use of Animals for Scientific Purposes, and within University of Adelaide guidelines for the use and care of laboratory animals.

### Generation and Maintenance of *Sgta*-Null Mice

Heterozygous *Sgta* knockout mice were generated by Ozgene Pty Ltd (Bentley, Aus.). The gene encoding *M. musculus Sgta* is located on Chromosome 10 and has 12 exons, 10 of which are protein coding. Structurally, SGTA exhibits a central tandem array of 3 TPR motifs, a glutamine-rich C-terminal domain and an N-terminal domain containing a potential short coiled coil motif (as reviewed in^1^). Our strategy was to delete the coding region of exons 4 and 5 of *M. musculus Sgta*. This approach would result in either: (1) cryptic splicing between exons 3–6, generating a stable mRNA coding for a truncated protein lacking the key functional domains, TPR1 and TPR2, for a functional knockout, or (2) a transcript lacking exons 4 and 5, potentially rendering it unstable and leading to a complete knockout.

A targeting vector was designed to introduce a conditional mutation into the mouse *Sgta* gene (Ensembl gene ID: ENSMUSG00000004937; NCBI Nucleotide Accession number: NM_024499) (Suppl Fig. 1C), employing mutant loxP sites to enable deletion of the loxP flanked (“floxed”) sequence under Cre recombinase-expressing conditions. LoxP sites were inserted into the introns flanking exons 4 and 5, incorporating TPR1 completely and part of TPR2 of *M. musculus Sgta.* The loxP site flanking exon 4 was inserted upstream and the loxP site flanking exon 5 was inserted downstream of a neomycin selection cassette (Phosphoglycerate Kinase (PGK)-neo). The selection cassette was flanked with Flippase Recognition Target (FRT) sites to enable removal by FLPe-mediated recombination. The targeting vector was inserted into a plasmid backbone PelleR (Ozgene) containing the PGK-neo cassette (Suppl Fig. 1C), linearised and electroporated into C57BL/6-derived embryonic stem cells (Ozgene). Correctly targeted stem cell clones were microinjected into recipient murine blastocysts, which were transferred into pseudo-pregnant foster mothers. Offspring were crossed with C57BL/6 mice to generate embryonic stem cell-derived targeted mutant progeny (heterozygous *Sgta* wt/flox), as confirmed by genotyping. These mice were crossed with OzCre mice, which express *Cre* knocked into the ubiquitously expressed *Gt*(*ROSA*)*26Sor* locus. Global tissue expression of Cre initiated loxP-mediated deletion of exons 4 and 5 of *Sgta* in early embryonic development and generation of *Sgta*^+/−^ +*Cre* mice. To minimise the impact of *Cre*-related toxicity^62^, *Cre* was eradicated by back-crossing *Sgta*^+/−^ *Cre* mice to C57BL/6 and it was only *Cre*-negative (Δ*Cre*) mice that were used subsequently and described herein.

Mice were housed under specific pathogen-free conditions on a 12-hr light/dark cycle at the University of Adelaide Medical School Animal House (Adelaide, Aus). Mice were group-housed (unless specified below) in individually ventilated cages in a temperature-controlled environment and were provided autoclaved standard rodent chow (Meat Free Rat and Mouse Diet, Specialty Feeds, Glen Forrest, Aus; 14.0 mJ/kg (23% and 12% energy from protein and fat, respectively, pre-autoclave)) and water *ad libitum*.

### PCR-based Genotyping

Confirmation of removal of the “floxed” *Sgta* sequence in mice was determined by PCR-based genotyping. Extraction of genomic DNA from tail or ear clippings was performed using the REDExtract-N-Amp Tissue PCR Kit (Sigma-Aldrich, St Louis, US) according to manufacturer’s instructions. A portion of this DNA was applied to PCR-based genotyping for the determination of the presence of (1) *Sgta* alleles (WT vs knockout) and (2) *Cre* (positive or negative).

1. Genotyping for the *Sgta* alleles employed 2 primer sets, referred to as ‘SGTA Knockout’ or ‘SGTA Intact’ primer pairs. The ‘SGTA Knockout’ primer pair was designed to yield a 389 bp product in response to a deleted *Sgta* allele (+/−, −/−) and a higher molecular weight product (450 bp) was observed in the presence of WT *Sgta* alleles. The ‘SGTA Intact’ primer pair was designed to yield a 765 bp product in response to an intact *Sgta* allele (WT, +/−) and the absence of a product in the presence of homozygous *Sgta* deletion (−/−). Genotyping primer sequences (5′-3′) were as follows, ‘SGTA Knockout’: Forward (FWD) CACAGTTGCCATCCAGTGTC and Reverse (REV) CTGGGGAGAGGAAACTGTCAGT; ‘SGTA Intact’: FWD TGACAGGACACCACCCTCTGAA and REV GGAGGGTCAGCCTGGGTTAAA.
2. Genotyping reactions for identifying the presence of *Cre* utilised a published primer pair^63^ that yields a 727 bp product in the presence of *Cre* and no product in its absence.

PCR conditions were an initial denaturation at 94 °C for 3 min; 34 cycles of 92 °C for 1 min, 58 °C for 1 min and 72 °C for 2 min; followed by final elongation at 72 °C for 10 min.

### *SGTA* mRNA in Normal Human Tissues and Gene Expression during Development in Mice

The distribution of *SGTA* mRNA in normal human tissue samples was assessed from publically available data through the Oncomine database ( https://www.oncomine.org/). *SGTA* mRNA content (log2 median-centred intensity) was measured using the Human Genome U133 Plus 2.0 Array on 504 and 353 normal human tissue samples for the Roth Normal and Roth Normal 2 cohorts, respectively^35^. A heat map was generated using conditional formatting and a colour scale based on the value in the cell and represented graphically, and categorised into tissues of endodermal, ectodermal and mesodermal embryonic origin (Suppl Fig. 1A). Mouse *Sgta* gene expression during embryonic development and in gametes was assessed using public data in Gene Expression Barcode 3.0 ( http://barcode.luhs.org/).

### Assessment of Fertility, Breeding Efficiency and Offspring Viability

All *Sgta*^+/−^ and *Sgta*^−/−^ breeding pairs were monitored for time to litter (date paired to date litter born) and pup yield was assessed for litter size, live born, stillborn (birth/d1) and neonatal death (postnatal d2-21). Offspring were reared by their mother until 3 wks-of-age, at which point they were removed from the breeding cage, weaned, sexed, tail-tipped (for genotyping), and placed in a cage with littermates of the same sex. Genotype distribution of litters was monitored for normal Mendelian inheritance in the case of *Sgta*^+/−^ breeders. Sex distributions were determined for all litters. From 3 wks, body weight was determined once-weekly.

### Birth Weight, Pup Body Weight and Food Intake

A cohort of offspring from *Sgta*^+/−^ and *Sgta*^−/−^ breeders were analysed for birth weight (d0), neonatal weight (d1-21) and for food intake or for body weight post-weaning (d21-105). To assess birth weight, a cohort of female breeders were left paired with males and checked daily 1–5 hrs after lights-on (7 AM), and any newborn pups were weighed. Pups were weighed daily and any dead pups collected and genotyped for SGTA status and sex. Upon weaning (strictly at d21), pups were individually housed and food intake assessed. During food intake assessment, food and water were provided *ad libitum*. From d 21-105, feed was first weighed, then placed in the cage for ~24 hrs whereupon the remaining feed and crumbs were weighed. Feed efficiency was calculated as weight gained from d21-105, relative to cumulative feed consumption for this time period (g weight gained/g feed consumed*100).

### Post-mortem Tissue Collection

A cohort of offspring from *Sgta*^+/−^ breeding pairs were assessed for phenotypic differences at adult (20 wks-of-age) and aged (60 wks-of-age) time points. From weaning (3 wks), body weight was measured weekly and on the day culled. At 20 or 60-wks-of-age, food was removed between 7–10 AM and mice were fasted for 5 hrs, with continued access to water. Post-fast, mice were humanely culled by carbon dioxide asphyxiation. Body weight and length (nose-to-tail base), and anogenital distance (AGD), were measured. Blood was drawn by cardiac puncture and serum separated using Minicollect tubes (Greiner Bio-One International, Kremsmünster, Austria). The following organs were rapidly dissected and weighed: in males, the whole genitourinary (GU) tract and subsequently dissected testes, vas deferens, epididymis, seminal vesicles and prostatic lobes (anterior, dorsolateral and ventral), penis and preputial gland; in females, the left and right abdominal mammary glands, whole GU tract and subsequently dissected uterus, ovaries and fallopian tubes; and in both males and females, the thymus, heart, lungs, pancreas, liver, spleen, stomach, intestine, colon, kidneys, brain and femur; adipose tissue depots (visceral mesenteric, pooled perirenal and retroperitoneal, gonadal (epididymal - males and periovariac - females) and interscapular brown); and skeletal muscles (gastrocnemius, tibialis, extensor digitorum longus and soleus). Male penis length was also assessed. In the case of paired organs, one of the pair was placed into RNA*later* (Ambion Inc, Austin, US) and the other in 10% neutral buffered formalin (NBF). Singular organs were halved and placed in RNA*later* or 10% NBF. Following incubation (4 °C, ~24 hrs), organs in RNA*later* were stored at −80 °C and those fixed in 10% NBF underwent overnight processing and were embedded in paraffin wax. Serum aliquots were stored at −80 °C.

### Serum Hormone Measurements

Serum from adult mice was assessed for levels of Insulin-like Growth Factor 1 (IGF-1) and Growth Hormone (GH) using the Mouse/Rat IGF-1 Quantikine ELISA (R&D Systems, Minneapolis, US) and Millipore Rat Mouse GH ELISA (EMD Millipore, St Charles, US), as specified by the manufacturer.

### Tissue Pathology, Immunohistochemistry and Immunofluorescence

Formalin-fixed paraffin-embedded tissue sections (4 μm) from adult and aged mice were stained with haematoxylin and eosin and assessed for tissue morphology by a pathologist (SJ). For immunohistochemical analyses, adult mouse tissue sections were incubated in 0.3% hydrogen peroxide (15 min) and antigen retrieval was performed by decloaker (10 mM citrate buffer (pH 6.5); 5 min at 125 °C and 10 sec at 90 °C). Cooled sections were subjected to successive 30 min blocking incubations in avidin/biotin block (Invitrogen, Mulgrave, Aus); Rodent Block M (Biocare Medical, Concord, US); and 5% goat serum. SGTA primary antibody-treated sections were incubated overnight (4 °C). SGTA immunoreactivity in normal WT mouse tissues was assessed using ‘full length SGTA’ ProteinTech antibody (11019-2-AP rabbit polyclonal; Chicago, US) and in *Sgta*^+/−^ offspring tissues by ‘N-terminal SGTA’ Santa Cruz antibody (sc-373978 mouse monoclonal; Dallas, US). Primary antibody omission was employed as a negative control. Immunogenic SGTA was visualised by a standard immunoperoxidase reaction using biotinylated anti-rabbit or anti-mouse antibody (1:400, Dako Aus Pty Ltd, North Sydney, Aus), streptavidin-horseradish peroxidase complex (1:500, Dako) and diaminobenzidine tetrahydrochloride. All sections were scanned using the NanoZoomer Digital Pathology image scanner (Hamamatsu Photonics, Hamamatsu City, Japan).

For immunofluorescent analyses, tissue sections from adult mice were subjected to blocking as above, except 10% goat serum was employed, and then incubated overnight (4 °C) in a solution containing SGTA (sc-373978) and AR (Santa Cruz, SC-816) primary antibodies. Primary antibody omission served as a negative control. Visualisation of immunogenic SGTA and AR were achieved using red Alexa Fluor goat anti-mouse-594 and green Alexa Fluor goat anti-rabbit-488 (Invitrogen), respectively. Sections were mounted (Prolong Gold anti-fade reagent with DAPI (Invitrogen) blue) and visualised by a Zeiss confocal microscope, ‘Zen’ software and Zeiss LDM700 with Lumen X-Cite Series 120PC camera.

### Western Blot Analysis

Protein was extracted from homogenised adult mouse brain (~100 mg brain; 350 μl RIPA buffer containing protease inhibitor cocktail (Roche Diagnostics, Indiana, US)) by centrifugation. Protein yield was determined by Bradford assay (Bio-Rad Laboratories, Hercules, US; bovine serum albumin (BSA) standard). Protein (80 μg) was subjected to gel electrophoresis utilising Criterion XT 1.0 mm gels (Bio-Rad) and transferred to nitrocellulose membrane (Hybond-C Extra 0.45 μm; Amersham Biosciences, Rydalmere, Aus). Membranes blocked in 3% BSA-TBS/T (Tris buffered saline (TBS)/0.1% Tween-20 (T)) overnight (4 °C) were probed for SGTA using ‘full length SGTA’ (ProteinTech), ‘C-terminal SGTA’ (Aviva BioSciences, San Diego, US) or ‘N-terminal SGTA’ (Santa Cruz) antibodies diluted at 1:1,000 (full, C-terminal) or 1:500 (N-terminal) in 1% BSA-TBS/T (60 min, room temperature). Immunoreactivity was detected using polyclonal goat anti-rabbit (1:1,000) or rabbit anti-mouse IgG/HRP (1:500) antibodies (Dako) followed by chemiluminescent visualisation (Super Signal West Dura Extended Duration, Thermo Fisher Scientific, Scoresby, Aus) and ChemiDoc MP imaging (Bio-Rad). Reference proteins used were actin (Santa Cruz Biotechnology, sc-1616, 1:2,000) or Histone H-3 (Abcam, ab7766, 1:500).

### Quantitative Real Time (qRT-) PCR

Total RNA was extracted from adult brain, ovary, mammary, anterior prostate and testis tissue. Tissues were homogenised in Buffer RLT (Qiagen, Germantown, US) and 2-Mercaptoethanol (Sigma-Aldrich) using a Precellys-24 homogeniser then passed through a QIAshredder column (Qiagen). Homogenate was then subjected to an RNeasy spin column workflow, as per Qiagen instructions. Eluted RNA quality and quantity were assessed (NanoDrop, Thermo Fisher Scientific), following which RNA (1 μg) was DNase treated (Ambion RNA TURBO DNA-free kit) and reverse transcribed (iScript First-Strand cDNA Synthesis Kit, Bio-Rad; 500 ng total RNA used as template). qRT-PCR primers for target genes of interest were designed using Vector NTI (Life Technologies) and synthesised by Sigma-Aldrich Pty Ltd (Sydney, Aus) (see Suppl Table 1). Triplicate qRT-PCR reactions were carried out using iQ SYBR Green Supermix and the CFX384 real-time PCR detection system (Bio-Rad). In each reaction, 2 μl cDNA, 0.5 μl each of FWD and REV primers (5 pm/μl) and 5μl SYBR Green mastermix (Applied Biosystems) were added to a final volume of 8 μl. PCR cycling conditions were 95 °C for 3 min, followed by 40 cycles of: 95 °C for 15 sec, annealing temperature (as described in Suppl Table 1) for 30 sec, and 72 °C for 30 sec; following which steps at 95 °C for 1 min, 55 °C for 1 min and 60–70 °C for 10 sec (3-step amplification and melt curve) were performed. In parallel, mRNA expression of a 5 candidate reference gene panel was determined and the 2 most stable (determined by geNorm analysis) were used to normalise target gene mRNA expression using the delta-delta cT method. PCR product specificity was validated with the use of a melt curve and by GelRed agarose gel electrophoresis. Reference genes utilised for normalisation were: ovary and anterior prostate – *Actb* and *Tbp*; mammary – *Tbp* and *Hprt1*; testis and brain – *Actb* and *Tfrc*.

### Statistical Analyses

All data presented as mean ± SEM of parameters. Shapiro-Wilk tests of normality and Levenes statistic tests of homogeneity of variance were performed and Q-Q plots assessed for all parameters. One-way ANOVA, with pairwise comparisons (Tukey or Dunnett’s T3 post-hoc), was used to determine the effect of genotype (WT, *Sgta*^+/−^, *Sgta*^−/−^) on mRNA expression. The effect of genotype, age (20, 60 wks) and sex (male, female) on body weight were assessed using a linear mixed model. Two-way ANOVA, with pairwise comparisons (Sidak or Dunnett’s T3 post-hoc), was used to determine the effect of genotype, age, sex and their interaction on the remaining parameters. If tests of normality failed, non-parametric Median tests were employed. Likewise, if data did not display homogeneity of variance, post-hoc tests in which equal variances are not assumed (Dunnett’s T3) were employed. Observed vs expected frequencies of genotype and sex were assessed by Chi-square analyses using GraphPad Prism ver.6.04 and linear regression analyses to determine relationships between variables (Pearson score (R) and *P* value) were performed using SigmaPlot ver.11; all other aforementioned statistics were performed using Statistical Package for Social Scientists ver.20. *P* < 0.05 was considered statistically significant.

## Additional Information

**How to cite this article**: Philp, L. K. *et al*. Small Glutamine-Rich Tetratricopeptide Repeat-Containing Protein Alpha (SGTA) Ablation Limits Offspring Viability and Growth in Mice. *Sci. Rep.* **6**, 28950; doi: 10.1038/srep28950 (2016).

## Supplementary Material

Supplementary Information

## Figures and Tables

**Table 1:** Reproductive Demographics of Heterozygous and Homozygous *Sgta*-deficient Breeding Pairs.

Breeder Statistics	*Sgta*^−/−^ x *Sgta*^−/−^	*Sgta*^+/−^ x *Sgta*^+/−^	Intact Comparator
Number of Breeding Pairs	6	15	6
Number of Litters	18	40	13
Average Litter Size (±SEM)	6.11 ( ± 0.58)^†‡^	7.33 ( ± 0.41)	7.54 ( ± 0.70)
Average Days to Litter (±SEM)	30.14 ( ± 3.54)	27.56 ( ± 2.18)	25.83 ( ± 2.75)
**Offspring Statistics**	****Sgta^−/−^ x *Sgta*−/−**	****Sgta^+/−^ x *Sgta*+/−**	**Intact Comparator**
Total Pups Born	110	293	98
Live Pups Born	104 (94.55%)	288 (98.29%)	96 (97.96%)
Stillborn Pups	6 (5.45%)	5 (1.71%)	2 (2.04%)
Neonatal Deaths	32 (30.77%)*^#^	39 (11.46%)	9 (9.38%)
Pups Weaned	72 (69.23%)*^#^	249 (86.46%)	87 (90.63%)
Male Pups Weaned	34 (47.22%)	132 (53.01%)	50 (57.47%)
Female Pups Weaned	38 (52.78%)	117 (46.99%)	37 (42.53%)

Statistics: ^#^*P* ≤ 0.05, trend ^†^*P* < 0.10, vs intact; *P ≤ 0.01, trend ^‡^*P* < 0.06, vs *Sgta*^+/−^.

**Table 2:** Growth Mediator and Androgen-Regulated mRNA Expression in the Prostate (PRO), Testis (TES), Mammary (MAM) and Ovary (OVA) Tissue of Adult *Sgta*-null Mice.

*Gene*	Males				Females			
	Tissue	WT	*Sgta*^**+/−**^	*Sgta*^**−/−**^	Tissue	WT	*Sgta*^+/−^	*Sgta*^−/−^
Growth Mediators								
*Ghr*	PRO	12.02 ± 0.98	10.21 ± 1.22	9.34 ± 1.32	MAM	4.44 ± 0.64	5.15 ± 0.57	4.93 ± 0.63
*Ghr*	TES	0.05 ± 0.004	0.05 ± 0.001	0.05 ± 0.002	OVA	2.42 ± 0.19^a^	3.16 ± 0.20^ab^	3.42 ± 0.33^b^
*Igf-1*	PRO	1.28 ± 0.11	1.16 ± 0.10	0.96 ± 0.09	MAM	3.84 ± 0.38^ab^	5.21 ± 0.62^a^	3.25 ± 0.60^b^
*Igf-1*	TES	0.28 ± 0.03	0.28 ± 0.02	0.28 ± 0.02	OVA	2.24 ± 0.17	2.54 ± 0.15	2.77 ± 0.16
*Igf-1r*	PRO	11.46 ± 0.81	11.67 ± 0.92	10.53 ± 0.86	MAM	4.22 ± 0.42	4.29 ± 0.19	4.63 ± 0.48
*Igf-1r*	TES	0.87 ± 0.07	0.77 ± 0.04	0.89 ± 0.05	OVA	3.56 ± 0.25	3.72 ± 0.22	3.68 ± 0.18
Androgen-Regulated								
*Hp-1*	PRO	0.07 ± 0.03	0.05 ± 0.02	0.02 ± 0.02	MAM	2.56 ± 0.31	3.39 ± 0.32	3.48 ± 0.56
*Hp-1*					OVA	0.39 ± 0.03	0.41 ± 0.03	0.42 ± 0.03
*Odc1*	PRO	0.35 ± 0.03	0.38 ± 0.03	0.34 ± 0.03	MAM	0.16 ± 0.01	0.17 ± 0.01	0.17 ± 0.01
*Odc1*	TES	0.93 ± 0.05	0.90 ± 0.04	0.94 ± 0.06	OVA	0.39 ± 0.03	0.41 ± 0.03	0.42 ± 0.03
*Rhox5*	TES	0.84 ± 0.05	0.77 ± 0.04	0.94 ± 0.09	OVA	0.89 ± 0.13	1.00 ± 0.15	1.04 ± 0.21

Means with different letters are significantly different, *P* < 0.05.
