# Supplementary material for: Small Glutamine-Rich Tetratricopeptide Repeat-Containing Protein Alpha (SGTA) Ablation Limits Offspring Viability and Growth in Mice
Source: Sci Rep. 2016 Jun 30;6:28950. doi: 10.1038/srep28950 (PMC4928056; doi:10.1038/srep28950)
Supplement: Supplementary Information [file srep28950-s1.pdf]

Small Glutamine-Rich Tetratricopeptide Repeat-Containing Protein Alpha (SGTA) Ablation Limits Offspring Viability and Growth in Mice

Lisa K Philp[1], Tanya K Day[1], Miriam S Butler[1], Geraldine Laven-Law[1], Shalini Jindal[1], Theresa E. Hickey[1], Howard I Scher[2], Lisa M Butler*[1,3], Wayne D Tilley*[1,3]

[1]Adelaide Prostate Cancer Research Centre and Dame Roma Mitchell Cancer Research Laboratories, Faculty of Health Sciences, University of Adelaide, Adelaide, Australia; [2] Memorial Sloan Kettering Cancer Center, New York, NY, USA; and [3] Freemason's Foundation Centre for Men's Health, School of Medicine, Faculty of Health Sciences, University of Adelaide, Adelaide, Australia. *Final authors had equal contribution.

Corresponding Author during Submission: Dr Lisa K Philp, Dame Roma Mitchell Cancer Research Laboratories, School of Medicine, University of Adelaide, DX Number 650 801, Adelaide, South Australia, 5000, Australia. Phone: +61 8222 3240, Fax: +61 8222 3217, Email: lisa.philp@qut.edu.au.

Corresponding Author of Final Manuscript: Professor Wayne D Tilley, Dame Roma Mitchell Cancer Research Laboratories, School of Medicine, University of Adelaide, Level 4 Hanson Institute Building, DX Number 650 801, Adelaide, South Australia, 5000, Australia. Phone: +61 8222 3252, Fax: +61 8222 3217, Email: wayne.tilley@adelaide.edu.au,

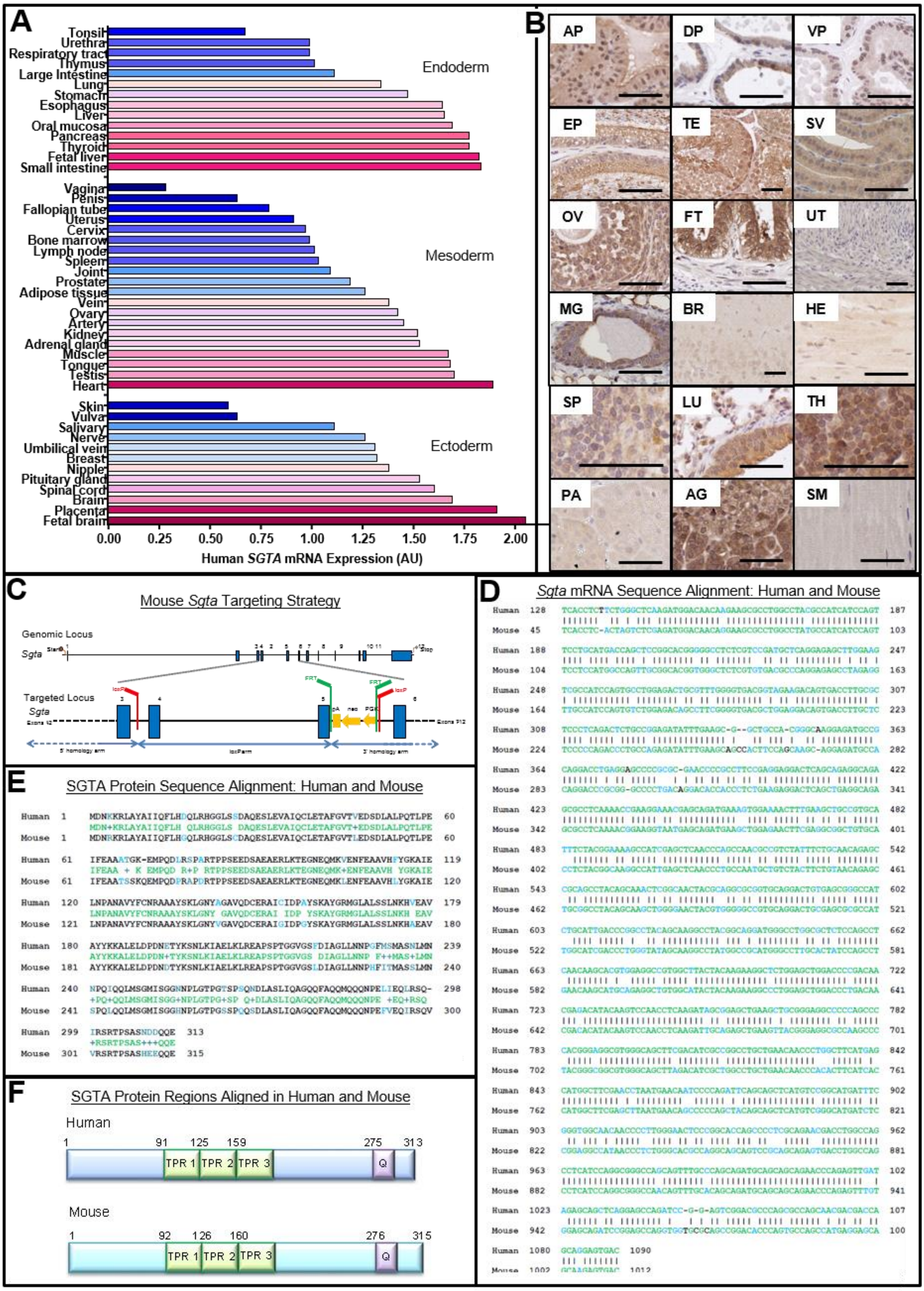

Suppl Figure 1[A-F]. Generation of *Sgta*-null Mice and Comparison of SGTA mRNA and Protein Sequence between *Homo sapiens* and *Mus musculus*. [A] *SGTA* mRNA expression in normal human tissues grouped by developmental origin (endo-, meso- and ecto-derm). **[B]** SGTA

immunostaining depicting broad protein expression in normal C57BL/6 mouse tissues [antibody dilution]: AP, anterior prostate [1:500]; DP, dorsolateral prostate [1:500]; VP, ventral prostate [1:500]; EP, epididymis [1:500]; TE, testis [1:500]; SV, seminal vesicles [1:1000]; OV, ovary [1:500]; FT, fallopian tubes [1:500]; UT, uterus [1:1000]; MG, mammary gland [1:1000]; BR, brain [1:2000]; HE, heart [1:500]; SP, spleen [1:500]; LU, lungs [1:500]; TH, thymus [1:500]; PA, pancreas [1:2000]; AG, adrenal gland [1:500]; SM, skeletal muscle [1:1000]. Scale bars represent 50µm. **[C]** Schematic representation of the targeting strategy employed to ablate mouse *Sgta*. **[D]** Sequence alignment of human and mouse *Sgta* mRNA. Green letters represent 83% identity (identical residues at the same positions in an alignment), black letters (1%) represent a gap, introduced to improve alignment, compensating for insertions and deletions and blue letters (16%) represent positive residues have preserved physicochemical properties. **[E]** Sequence alignment of human and mouse SGTA protein, green letters represent 88% identity, teal letters represent 94% positives, blue letters and a gap in the central sequence represents dissimilar residues and black letters and a gap in the central sequence represents an inserted gap (0%). **[F]** Alignment of functional SGTA protein regions in human and mouse: TPR, tetratricopeptide repeat; Q, glutamine-rich domain.

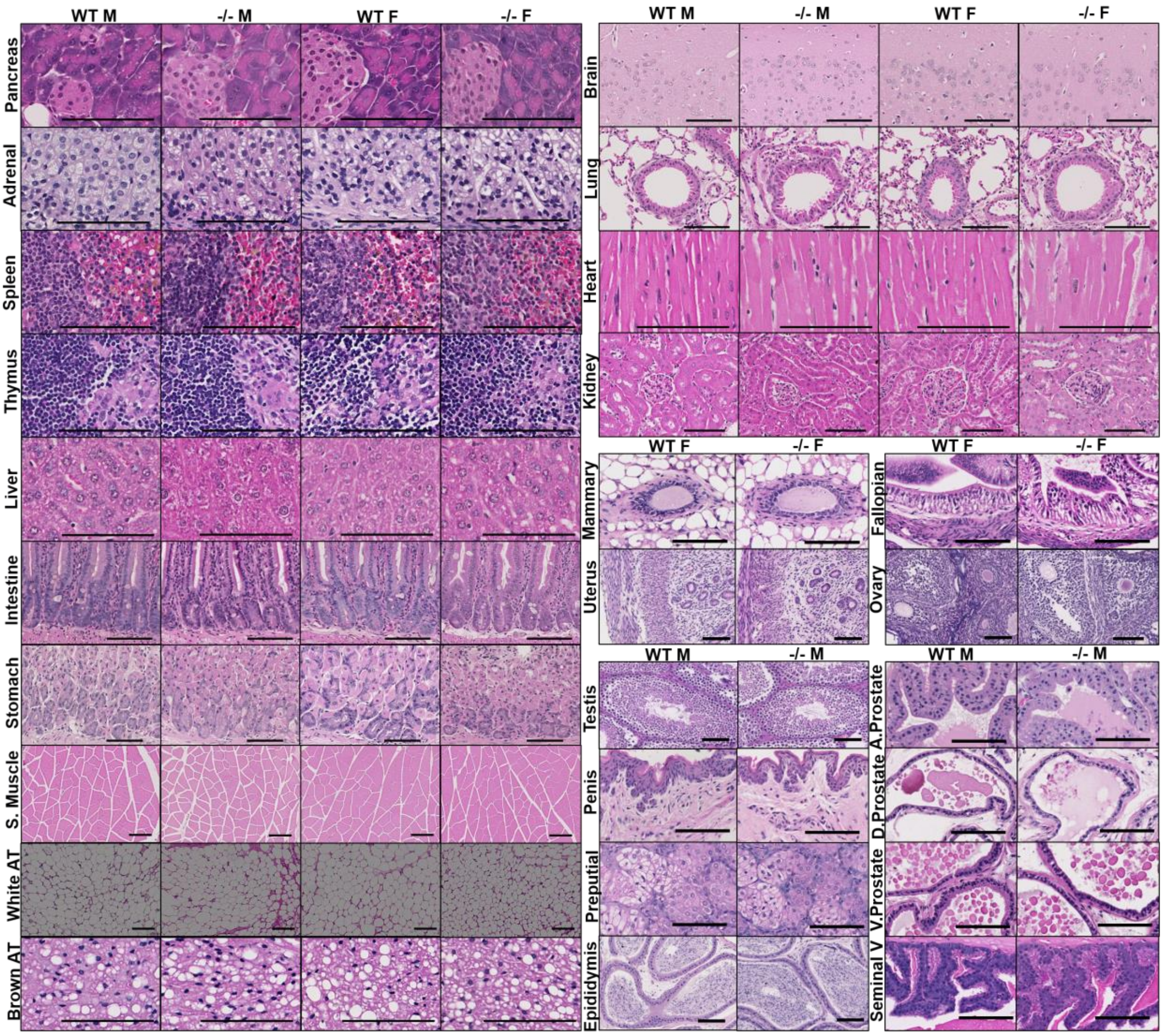

Suppl Figure 2. Cell Morphology and Pathology of Adult Male and Female Wild-type (WT) and *Sgta*-null (-/-) Organs. Haematoxylin and Eosin stained tissue sections of the pancreas, adrenal gland, spleen, thymus, liver, intestine, stomach, skeletal muscle (S. muscle, gastrocnemius muscle), white adipose tissue (White AT, perirenal-retroperitoneal fat pad), brown adipose tissue (Brown AT, interscapular brown fat pad), brain, lung, heart, kidney, in male (M) and female (F) mice; Haematoxylin and Eosin stained female tissues, including the mammary, uterus, fallopian tube and ovary; and male tissues, including the testis, penis, preputial gland, prostate (A, anterior lobe; D, dorsolateral lobe; V, ventral lobe) and seminal vesicles (Seminal V). All scale bars represent 100μm.

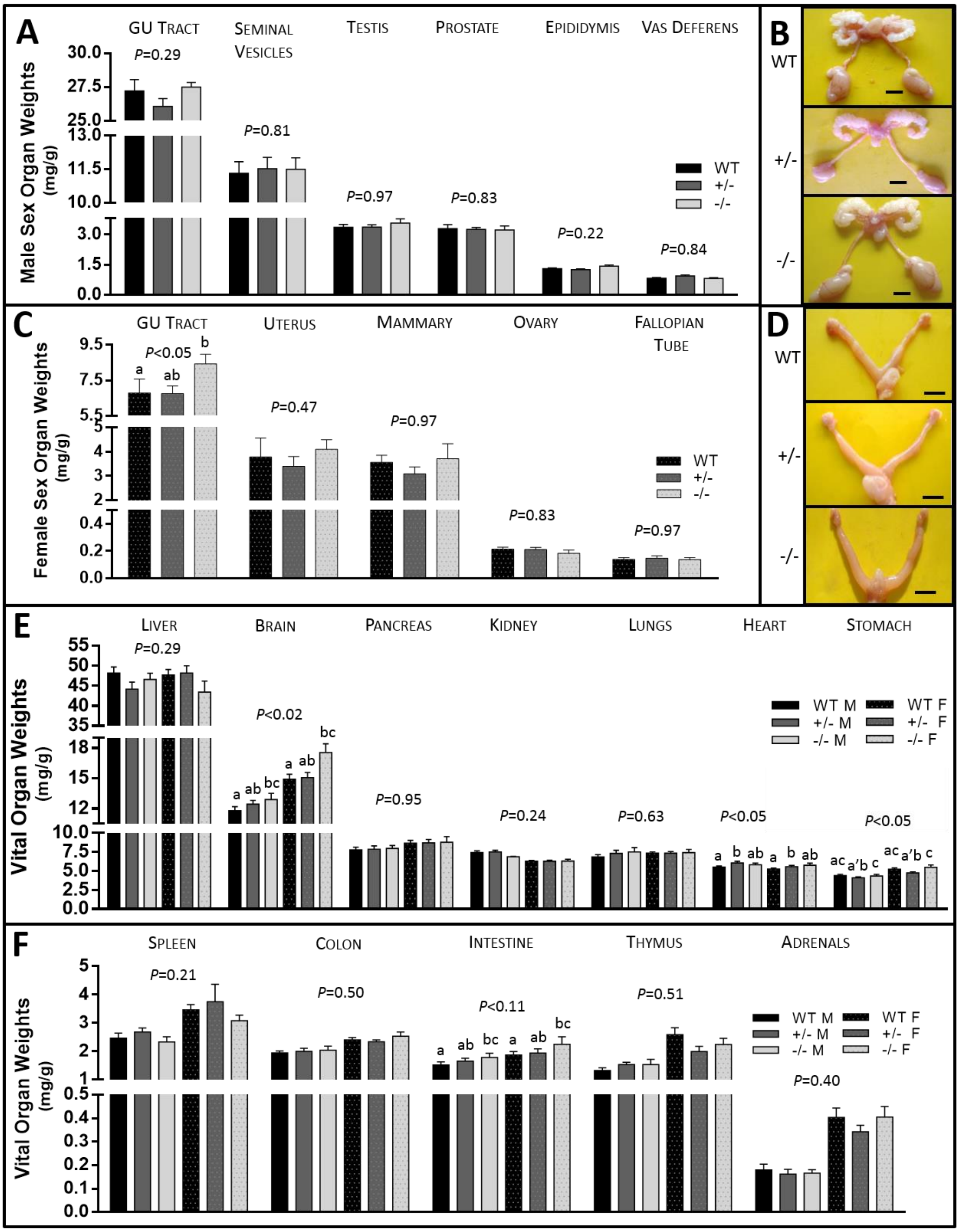

Suppl Figure 3. Weights of Reproductive and Vital Organs in Adult *Sgta*-deficient Mice.

[A]. In male (M) mice, relative weight (mg/g body weight) of the whole genitourinary (GU) tract, seminal vesicles, testis, prostate, epididymis and vas deferens (n= 7-10/group, except prostate n=12-15/group). WT, wild-type, +/-, *Sgta*$^{+/-}$ and -/-, *Sgta*$^{-/-}$. **[B]** Representative male genitourinary tract relative to genotype. Scale bar = 5 mm. **[C]** In female (F) mice, relative weight (mg/g body weight) of

the whole genitourinary (GU) tract, uterus, mammary, ovary and fallopian tube (n = 8-9/group GU, uterus; n=8-14/group ovary, mammary, fallopian tube). Means with different letters are significantly different, $P<0.05$. **[D]** Representative female genitourinary tract relative to genotype. Scale bar = 5 mm. **[E]** Relative weight (mg/g body weight) of vital organs, the liver, brain, pancreas, kidney, lungs, heart and stomach (n=7-9/group). Means with different letters are significantly different, $P<0.05$; a' trend, $P<0.06$, +/- vs WT. **[F]** Relative weight (mg/g body weight) of more vital organs, the spleen, colon, intestine, thymus and adrenals (n=7-10/group). Means with different letters are significantly different, $P<0.05$.

Suppl Table 1. *Target and Reference* Gene Primer Sequences (Forward, FWD; Reverse, REV) utilised for mRNA Expression Analyses by qRT-PCR and their Optimised Annealing Temperature.*

Gene Symbol & Accession #	***FWD Sequence (5'-3')***	***REV Sequence (5'-3')***	***Annealing Temp (°C)***
Ar *(NM_013476)*	TCCCAGTCCCAATTGTGTCAA	TCGATGGGTAAAACATGGTCC	58.0
Dnajc7 *(NM_019795)*	AATGGCGGCATGTCGTAGTT	CATGACGGCATTGGCGTT	58.0
Fkbp4 *(NM_010219)*	GAGTGGACATCAGCCCCAAA	CAAAGACTCGGTCCCCGAT	62.5
Fkbp5 *(NM_010220)*	CACGACATTCCGATTGGGAT	CCAAACTTAGGCTTCCCGG	61.5
Ghr *(NM_010284)*	CTGCAAAGAATCAATCCAAGCC	CAGTTCAGGGGAACGACACTT	60
Hp-1 *(NM_017370)*	GCTATGTGGAGCACTTGGTTC	CACCCATTGCTTCTCGTCGTT	67
Igf-1 *(NM_184052)*	CACTCATCCACAATGCCTGT	TGGATGCTCTTCAGTTCGTG	60.5
Igf-1r *(NM_010513)*	CATGTGCTGGCAGTATAACCC	TCGGGAGGCTTGTTCTCCT	60.5
Odc1 *(NM_013614)*	GACGAGTTTGACTGCCACATC	CGCAACATAGAACGCATCCTT	61.1
Ppid *(NM_026352)*	ACCGGGAAGCCTCTCCATT	TTCGCCACCTGTCCCATTC	59.0
Ppp5c *(NM_011155)*	TCAAGACACAGGCCAACGACTA	ATTGCCGGGGTTCAACTCGAT	63.0
Rhox5 *(NM_008818)*	CATTTTGCAGCGCACTAATTCC	AGCCCTCCTGATCTTAAACCA	61.5
Sgta Exon1-3 *(NM_024499)*	GTCTTCGGTCGACTGAGAGAT	GCCATGGAGGAACTGGATGA	60.0
Sgta Exon 4-5 *(NM_024499)*	GACAGGACACCACCCTCTGAA	TTGCCGTAGAGGTGCACAGC	60.0
Sgtb *(NM_144838)*	GGCTGAGGTTTGAGATGGCTA	CATCATCCCTGACATTAGCTGC	61.5
Stub1 *(NM_019719)*	TACACTGCCACAAGTGGGTT	GCGCAAGAGCTCAAGGAG	57.4
Actb* *(NM_007393)*	GGCTGTATTCCCCTCCATCG	CCAGTTGGTAACAATGCCATGT	64.5
Gapdh* *(NM_017008)*	TGCCGCCTGGAGAAACCTG	CTGTTGAAGTCACAGGAGAC	58.0
Tbp* *(NM_013684)*	CCTTGTACCCTTCACCAATGAC	ACAGCCAAGATTCACGGTAGA	60.0
Tfrc* *(NM_011638)*	GGACTGTAGAGGCGCTTCCTA	GGGTGTATGACAATGGTTCCC	60.0
Hprt1* *(NM_012583)*	CCTTGACTATAATGAGCACTTC	GCCACATCAACAGGACTC	56.4
